# National trends in the initial diagnosis and management of carpal tunnel syndrome: results from the ELECTS (ELEctrophysiology in Carpal Tunnel Syndrome) study

**DOI:** 10.1308/rcsann.2022.0087

**Published:** 2022-11-30

**Authors:** HH Chong, A See, K Kulkarni, ELECTS Collaborators

**Affiliations:** ^1^ University Hospital of Leicester NHS Trust, UK; ^2^ Kettering General Hospital NHS Foundation Trust, UK; ^3^ Pulvertaft Hand Centre, University Hospitals of Derby and Burton NHS Foundation Trust, UK

**Keywords:** Nerve conduction studies, Carpal tunnel syndrome, Median neuropathy, Electrophysiology

## Abstract

**Introduction:**

The optimal role of nerve conduction studies (NCS) in management of carpal tunnel syndrome (CTS) is unclear, with no standardised guidance. This study aimed to identify variation in practice in the initial diagnosis of patients with suspected CTS, alongside evaluating how NCS findings influence clinical decision making.

**Methods:**

A national multicentre collaborative survey was conducted in 2021. All centres providing surgery for CTS were invited to participate, primarily via social media. All middle–senior grade orthopaedic/plastic surgeons and advanced care practitioners that regularly manage new referrals for suspected CTS were eligible to respond. Local representatives at each participating site submitted their responses to a central team who collated and analysed the results.

**Results:**

A total of 137 healthcare professionals responded from 18 UK NHS Trusts. Of these 137, 124 (91%) reported not employing any validated clinical questionnaires in their routine practice, preferring to rely on clinical diagnosis and/or NCS if available, whereas 84 (61%) utilised NCS to aid diagnosis, with significant differences among professionals with differing experience (*p* < 0.01). The most common methods for determining the severity of CTS were history, examination and NCS. In symptomatic CTS with confirmatory NCS, over 50% of clinicians would choose surgical decompression as their first-line intervention. In cases of either negative NCS or atypical presentation, 37% and 51%, respectively, would consider conservative management (e.g. splintage) or steroid injection first line.

**Conclusions:**

With growing waiting lists for NCS and surgery, national consensus guidelines should be developed to support decision making, while maximising efficient utilisation of increasingly constrained resources.

## Introduction

Carpal tunnel syndrome (CTS) is one of the commonest compressive neuropathies, with a UK prevalence ranging from 7% to 16%.^1-5^ Nerve conduction studies (NCS) can be used to obtain an electrophysiological diagnosis of the site of compression, e.g. when there is clinical doubt, alongside enabling stratification of severity of the nerve compression.^2,6,7^ Although NCS have been shown to have both high sensitivity and specificity, negative NCS findings do not necessary exclude potential CTS.^8^

The precise role of NCS in diagnosing CTS remains debatable. The American Academy of Orthopaedic Surgeons (AAOS) 2016 guideline concludes limited evidence in the use of hand-held NCS in diagnosing CTS.^9^ In the UK, regional clinical commissioning group (CCG) guidelines vary widely, with some regions requiring a positive NCS before the offer of carpal tunnel decompression (CTD), whereas others do not.^10^ The National Institute for Health and Care Excellence (NICE) advocates that NCS are not necessary if typical symptoms are present, but should be considered if there is diagnostic uncertainty or if referral for surgical management is being undertaken.^11^ Similarly, the British Orthopaedic Association (BOA) commissioning guide does not recommend NCS in primary care, but states that NCS should be used in secondary care where there is either diagnostic uncertainty, or in cases of persistent/recurrent CTS.^9^

The aim of this study was to assess secondary care practises in the initial diagnosis of new patients with suspected CTS, particularly with regard to the use of electrophysiology, and where this investigation fits into the overall assessment algorithm alongside other available tools such as history, examination findings and other modalities.

## Methods

A multicentre survey study was conducted across UK secondary care centres between February and April 2021. All collaborators were invited via a trainee-led collaborative research network, with surveys circulated via trainee networks and social media.

### Survey

A survey was developed to assess current practices in the diagnosis and management of CTS. The objectives were to determine standard practise in managing new CTS referrals from primary care services, focusing on electrophysiology but also including the use of validated clinical questionnaires and imaging in diagnosis and severity stratification. To provide clinical context, four clinical were developed to investigate preferred management priority between non-operative measures (observation, splinting), steroid injection or CTD.

• Scenario 1 (clinically and NCS positive): clear clinical features, persistent motor/sensory loss, confirmatory positive electrophysiology.
• Scenario 2 (partially clinically and NCS positive): clear clinical features, no motor/sensory loss, confirmatory positive electrophysiology.
• Scenario 3 (only clinically positive): clear clinical features, negative/unsupportive electrophysiology.
• Scenario 4 (only NCS positive): unclear/intermittent/mild clinical features, positive electrophysiology (confirming median nerve compression).

The scenario questions were piloted with the first cohort of respondents to ensure reliability with the Cronbach alpha test. In total, the survey consisted of 14 questions, with mixed multiple choices and free text to allow adequate meaningful information collection without overburdening respondents (Appendix 1).

### Survey distribution

Collaborators were recruited in different hospital sites to coordinate the local distribution of survey across clinical teams using either standardised paper or electronic forms. All secondary care healthcare professionals that review new referrals for suspected CTS were invited to participate, including senior orthopaedic and plastic surgeons (consultants, middle grade or specialty registrars) and advanced care practitioners (ACPs, including extended scope therapists and specialist nurse practitioners). Responses were collated centrally and anonymised. Respondents consented to the use of their responses for publication.

### Statistical analysis

Results were analysed using IMB Statistical Product and Service Solutions (SPSS) Version 26 to produce baseline characteristics of demographic variables. Categorical variables were expressed as proportions or percentages. Free-text responses were classified under similar groups. No direct comparisons were made between regions. A chi-squared analysis, with a significance level of 0.05, was used to review the influence of clinical experience on the management of new CTS cases. The Cronbach alpha test was also performed to assess the reliability of the scenario questions.

## Results

### Demographics

In total, 137 survey responses were received (breakdown by Hospital Deaneries in Appendix 2). Of these 137, 72 (53%) were from teaching hospitals and 65 (47%) from district general hospitals; 107 (78%) respondents were from an orthopaedic background. The breakdown of responses according to clinical role is listed in Table 1.

**Table 1:** Clinical role of respondents

Clinical role	Responses (*n*)	Percentage (%)
Orthopaedic surgery		
Consultant (hand specialist)	36	26
Consultant (non-hand specialist)	16	12
Specialty registrar/middle grade	55	40
Plastic surgery		
Consultant (hand specialist)	11	8
Consultant (non-hand specialist)	2	2
Specialty registrar/middle grade	7	5
Advanced care practitioners		
Physio and occupational therapists	6	4
Advanced nurse practitioners	4	3

### Use of clinical questionnaires

**Figure 1:**
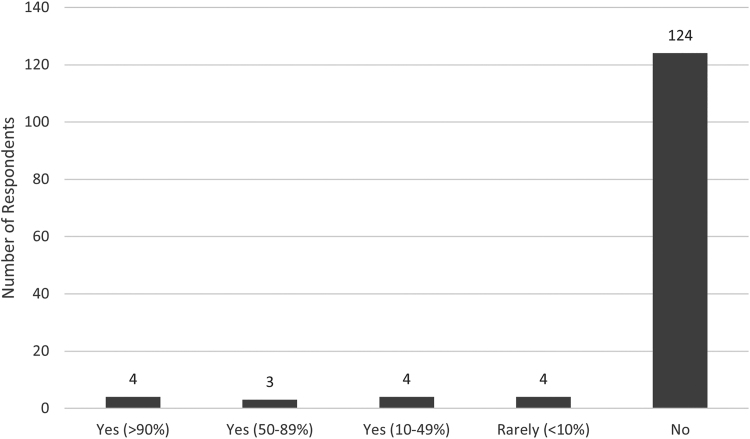
Use of clinical questionnaires in the diagnosis of CTS. Most (89%) do not use validated clinical questionnaires. Only 11% routinely used clinical questionnaires and, of these, the frequency of use varied from <10% to >90% cases.

Validated questionnaires, including Kamath and Stothard, I-HaND Scale Version 2, Dash/Quick-Dash, Patient Evaluation Measure (PEM), EQ5D, Visual Analogue Scale (VAS), Michigan Hand Questionnaire and the Boston Questionnaire were used by only a minority of respondents (Figure 1). Of the cohort that routinely used questionnaires, four (31%) felt the findings might guide their management in the majority (>50%) of cases, whereas nine (69%) felt it was of less frequent benefit (two did not respond). Of the cohort that did not routinely use questionnaires, 67/122 (55%) felt a diagnosis should be made clinically, 22/122 (18%) preferred to rely on other modalities (mostly NCS) and the remainder cited other reasons, including lack of experience/incorporation into local practice/clear rationale, with some suggesting that screening questionnaires should ideally be performed by primary care teams before referral.

### Use of NCS

**Figure 2:**
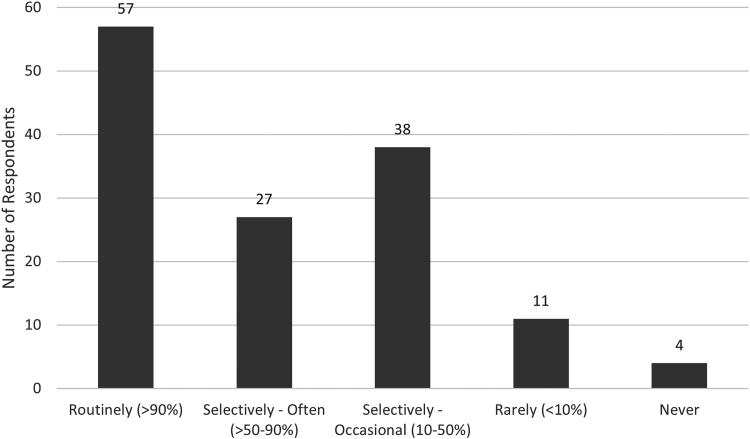
Use of NCS in the diagnosis of CTS. Most do use NCS for newly suspected CTS, but frequency of ordering CTS varies. Of those who routinely used NCS, 48 requested the study after clinic whereas 9 requested before first clinic. Chi-squared analysis found that specialty registrars and middle grade doctors were more likely to use NCS routinely, whereas hand surgeons (both plastics and orthopaedics) used NCS more selectively (*p* < 0.01).

Most respondents routinely utilised NCS in their practice to guide the diagnosis and management of newly suspected primary CTS (Figure 2). A total of 11 (8%) reported that their local referrals were always made with NCS already performed/requested, 88 (64%) reported this was sometimes the case, and 38 (28%) stated this was never the case. The rationale of clinicians that utilised NCS selectively or rarely is summarised in Table 2.

**Table 2:** Rationale of selective or rare utilisation of NCS in newly suspected CTS

Responses	*n*
Diagnostic uncertainty/atypical clinical presentation	55
Severe Disease	14
Diabetic/potential other neuropathy /possible secondary cause	15
Medicolegal purposes	5

CTS = carpal tunnel syndrome; NCS = nerve conduction studies

### Severity stratification and case scenarios

**Figure 3:**
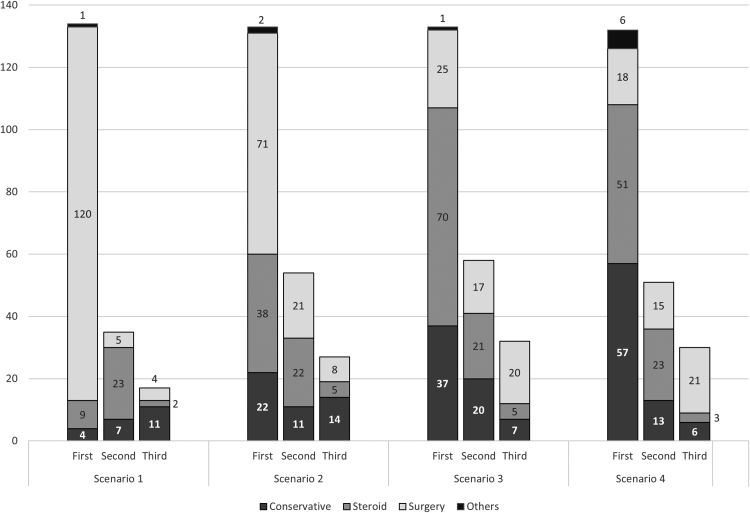
Preferred management in four hypothetical clinical scenarios In both scenarios 1 and 2 (clinically and NCS positive for primary CTS), most would advocate CTD first line. In scenarios 3 and 4 (clinical findings and NCS findings inconsistent), most propose more conservative initial management. No statistical correlation between clinical role/grade and management option of each scenario.

The importance of a basic history and examination in were emphasised in the approaches to stratifying CTS severity, summarised in Table 3. The scenarios questions were piloted on the first 15 respondents (equal mix of clinical roles), with the Cronbach alpha reliability test concluding an acceptable reliability of 0.7. Findings are summarised in Figure 3 and Table 4.

**Table 3:** Factors considered in severity stratification of newly diagnosed CTS

Factor	*n*
History	132
Examination	133
Questionnaire	5
NCS	115
Imaging	7
Others (e.g. quality of life, age)	4

CTS = carpal tunnel syndrome; NCS = nerve conduction studies

**Table 4:** Clinical scenarios: preferred management

Scenario 1 (clinical and NCS positive): clear clinical features, persistent motor/sensory loss and confirmatory positive electrophysiology				
**Management**	**Conservative (*n*)**	**Steroid injection (*n*)**	**Surgery (*n*)**	**Other (*n*)**
1st choice	4	9	120	1
2nd choice	7	23	5	0
3rd choice	11	2	4	0
Scenario 2 (partial clinical and NCS positive): clear clinical features, no motor/sensory loss and confirmatory positive electrophysiology				
**Management**	**Conservative (*n*)**	**Steroid injection (*n*)**	**Surgery (*n*)**	**Other (*n*)**
1st choice	22	38	71	2
2nd choice	11	22	21	0
3rd choice	14	5	8	0
Scenario 3 (only clinically positive): clear clinical features, but negative/ unsupportive/ lack of electrophysiology				
**Management**	**Conservative (n)**	**Steroid injection (*n*)**	**Surgery (*n*)**	**Other (*n*)**
1st choice	37	70	25	1
2nd choice	20	21	17	0
3rd choice	7	5	20	0
Scenario 4 (only NCS positive): unclear/intermittent/mild clinical features, but positive electrophysiology confirming median nerve compression				
**Management**	**Conservative (*n*)**	**Steroid injection (*n*)**	**Surgery (*n*)**	**Other (*n*)**
1st choice	57	51	18	6
2nd choice	13	23	15	0
3rd choice	6	3	21	0

NCS = nerve conduction studies

#### Alternative investigations

Of the 137 respondents, 81 (59%) utilise a diagnostic steroid injection when the diagnosis is unclear or atypical, for therapeutic management in selected cases or for prognostication, and 48 (35%) utilise imaging (USS or MRI) in either selected unclear cases, or when alternative or coexisting diagnosis are suspected (e.g. cervical radiculopathy, secondary causes of CTS such as tumour or following trauma).

## Discussion

This multicentre study highlights the wide variation in UK practice in the diagnosis and management of primary CTS.

There remains uncertainty regarding the value of electrophysiology in guiding decision making in new cases of suspected primary CTS, consistent with the findings of other authors.^10^ Although most (97%) respondents use NCS, rationale differed, although diagnostic uncertainty remained the primary reason (62%). Junior clinicians were more likely to use NCS, perhaps due to a lack of clinical experience resulting in a greater need for supportive diagnostic confirmation. In contrast, hand surgeons were more likely to use NCS selectively, placing greater emphasis on clinical history and examination for clinically straightforward cases.

Questionnaires have been demonstrated to be valid and reliable tools for measuring functional deficits secondary to CTS (carpal tunnel questionnaire, CTQ), alongside having high (90%) positive predictive value—comparable with NCS (92%)—for predicting symptomatic improvement (Kamath and Stothard), albeit not diagnosis.^12,13^ However, most respondents did not routinely use a questionnaire, with those that did suggesting it rarely changed their management.

When faced with a clear clinical presentation and positive (supportive) NCS (alongside non-operative measures being exhausted before referral), most clinicians would opt for CTD as their first-line management. When the clinical presentation is inconsistent with NCS findings, most clinicians opt for more conservative management, with either a trial of splinting or steroid injection. It is important to note that patients with a clear clinical picture of CTS but negative electrophysiology still benefit from CTD, hence the absence of supportive NCS should not preclude CTD.^14^ Gunnarsson *et al* suggested that an adequate clinical examination has equivalent sensitivity and specificity (94% and 80%, respectively) to neurophysiological examination (85% and 87%, respectively).^15^ Other available literature also advocates that provocative clinical tests have sufficient sensitivities and specificities, implying that NCS are unnecessary for straightforward cases and should be reserved for cases with diagnostic uncertainty, particularly given growing resource constraints.^16,17^

Although only 59% of our respondents reported using a steroid injection in the diagnosis of clinically uncertain CTS, as a low-cost combined diagnostic and therapeutic option, this could initially be utilised more frequently in place of NCS—particularly given the evidence that patients experiencing symptomatic relief of >3 months have better outcomes following subsequent CTD.^18,19^ However, it is important to note that, in a direct comparison, CTD was shown to produce better outcomes than steroid injection alone in idiopathic CTS.^20^

This study had several limitations. With most respondents being either non-consultant grade or non-specialist (hand surgeon), and with a limited dataset from only a proportion of England-based NHS Trusts, our data will not necessarily represent the breadth and gold standard of UK specialist practice. However, our results are therefore likely representative of ‘real-world’ differences in practice, with junior clinicians mirroring their supervising consultants, alongside their decision-making being influenced by a broader range of senior clinicians they have trained with across a larger number of rotational regional units. We also did not survey primary care clinicians, who increasingly manage CTS in community settings and who may adopt non-operative measures more frequently than their secondary care colleagues given the greater numbers of new presentations they encounter; this should form the basis of a future study, given the growing numbers managed in this setting.

## Conclusion

In summary, there is wide variation in diagnostic approaches to new cases of suspected CTS. Clear national evidence-based guidelines, influenced by the experience of relevant societies (including the British Society for Surgery of the Hand and BOA), are required to standardise management across the UK to improve patient care and optimise the use of increasingly constrained resources.
